# On Morbid Poisons

**Published:** 1851-03

**Authors:** John Simon

**Affiliations:** Surgeon to St. Thomas’s Hospital, etc.


*On Morbid Poisons.* By John Simon, Esq., F. R. S., Surgeon to St. Thomas's Hospital, etc.—By *morbid poison* we understand a product, which is the supposed specific cause of certain specific diseases; of syphilis, for instance; of scarlatina, of typhus, of glanders, of small-pox, of hydrophobia, and the like;—a product, which has many striking differences from all other poisons, but chiefly these: first, that while other poisons (oxalic, or hydrocyanic acid, or sulphuretted hydrogen gas) act directly in proportion to their dose, becoming more or less deadly in proportion as more or less of them is brought to bear on the organism; you may observe, contrariwise, that the *morbid* poison (the poison of contagion) produces its characteristic results, when given in the minutest conceivable doses, just as surely, and just as deadly, as when the system is saturated with it:—and secondly, that while common poisons diminish from the body, or at the most remain stationary, during the production of their effects, morbid poisons apparently undergo, within the body on which they act, a striking and singular increase.

The phenomena which follow infection with a morbid poison consist of certain local changes, attended by a peculiar constitutional state. The *local* changes may be generalised as sub-acute inflammatory processes, attended (perhaps preceded) by the deposition of a *specific material*, which material in most cases contains an agent capable, by inoculation, of producing in another person the same symptoms as have attended its own generation in the original sufferer. The peculiar *constitutional* state is one essentially of depression; modified no doubt, and intermixed with those phenomena of reaction which the living body (like a spring) always opposes to the direct pressure of exterior influences.

Of the local charges, partaking of an inflammatory character, our memory can give many illustrations: such [illegible] in the pustules of small-pox; in the cynanche and erythema, and kidney affection of scarlatina; in the intestinal ulcers of typhus; in the catarrh and eruption of measles; in the rupia or periostitis of syphilis; in the swollen parotid of mumps; in the dysentery of malarious fever; in suppurating tumors of glanders; and in various other symptoms that might be quoted.

And it is because of these local differences in *effect* that we are impelled to distinguish the *causes*, and to speak of them as *specific:* syphilis never produces ulcers in the ileum, scarlatina never causes iritis; the causative poison of the one disease differs from the causative poison of the other, for on the self-same subject it produces different effects.

[But there is probably a *material* in the system, or in the blood, upon which the morbid poison may act.]

What this material—the principle of infective disorders in the human subject—may originally have been, we are totally unable to say, but whatever may have been its first method of generation, we can now confidently speak of it as a possible product of the human body; we know that it is liable to develope itself out of some constituent of the human blood.

What are these constituents? Observation and argument sufficiently show, that the blood corpuscles and albumen can hardly be the constituents in question: first, because, after death by zymotic disease, they are found without evident alteration, and no considerable change in them could escape notice; secondly, because they are indispensable to life, and even their temporary transformation (if complete) would of necessity be fatal; thirdly, because immunity could never be attained by one attack of any particular disease, if it were requisite to exhaust these products: re-exposure to infection would ensure a return of the disease, and a reappearance of its phenomena.

For somewhat similar reasons, we may conclude, that the salts are not the elements concerned. Fibrine and the so-called extractive matters are what remain—can these be the ingredients in question? Substituting for the chemical phrase "extractive matters" the physiological one "waste of the tissues," I am strongly disposed to think an affirmative answer to that question; or, at all events, unhesitatingly to point here as the direction in which accurate pathological investigation may be made with most prospect of success.

For, in the first place, they are matters already in progress of decay, and therefore eminently susceptible of new modification; in the second place, they are unessential to the nutritive processes, and that removal of them from the system, which would give immunity from re-infection, might be accomplished without withdrawing a vital ingredient from the blood; in the third place, only of such matters as these can it be said, that some of them occur but once in life. In infancy, in early age, and till puberty, there are certain waste materials which never afterwards occur; the temporary cartilages have to waste away, the thymus gland has to decay, peculiar changes referable to the sexual system have to be accomplished, and the effete products of these changes have to be eliminated from the system. And fourthly, notice that the surfaces and organs most prone to affection in the diseases in consideration are those which are eliminative and defecating; those whose normal products can hardly be retained for any time within the body, much less out of it, without undergoing a fœtid decomposition, which sufficiently stamps them with an excrementitious character. Bowels, skin, kidney, tonsils, are the favorite resorts of the several fever poisons, just as they are the surfaces by which naturally the organic waste of the several tissues is eliminated. And it may not be amiss to notice that, whereas the normal and healthy discharge of these substances commonly tends to occur in the highest attainable form of oxidation; and whereas, under a variety of atmospheric circumstances interfering with their efficient oxidation, they must tend to accumulate in forms more susceptible of fœted decomposition; so it is peculiarly under such circumstances—where ventilation is defective—where human beings are unduly crowded—where the air is loaded with de-oxidizing influences—that zymotic diseases tend to affect the system either through a new generation of their poison, or through some vast increase of susceptibility thus engendered.

On enquiry, it might appear that the relations of infective material to these natural products are definite and constant; that one—let us, for instance, say syphilis—would stand in the particular relation to fibrin; it would be obvious that such an one would be of almost universal inoculativeness, and could only for a very short time, if at all, exhaust the patient's susceptibility to re-infection; and that a drug having certain relations to fibrin (mercury for example) would interfere with the affinities established by the disease. It might appear that another material, having its origin in the organic waste of nervous substance, would constitute the liability—say, to typhus; such an origin would almost fix the circumstances increasing our proneness to that disease, as well as prefigure the symptoms attending it. Of another material it might appear that it originates in the infantile decay of temporary cartilage, or of thymus, a decay occurring only once in life; that such material would constitute the susceptibility to measles or hooping-cough, a single attack of which commonly exhausts the patient's susceptibility for ever. Of a fourth material it might appear, that it arises in those changes of blood which attend the inflammatory and reparative processes, under direct atmospheric influence (as in open wounds, cutaneous or mucous,) and that in such a product would consist the humoral liability to erysipelatous infection, and to puerperal fever. I need not multiply these hypothetical cases, but, before leaving them, let me beg you to understand, that I employ them only as *illustrations;* that I do not adduce them as pictures of what occurs, only as diagrams explanatory of my meaning.

That the specific materials of the several morbid poisons, as they now pass daily under our notice, constituting the principles of zymotic infection, are either actually derived from the blood, or might have been thus derived, is quite a certainty. Whether each of them, in its first and original derivation, was a native ingredient of the blood, identical with that on which we now see its influence exerted; and whether its first conversion into a specific *materies morbi* occurred without exterior infection, are points which cannot be decided with confidence. In respect of many infected diseases, however, this view of their having first of all arisen spontaneously, would seem consistent with analogy. Experience confirms this theoretical view; for we not unfrequently hear of an outbreak of small-pox or scarlatina, where no communication can be traced with a person previously iufected; and we constantly have cases of typhus arising sporadically, where we may fairly consider the patient to have originated the disease within the limits of his own organization.

It is too much the custom to speak of the personal predisposition to infective disorders, as though it consisted in a condition of mere debility. There is no foundation for this view. If we examine cases of pure debility—such as occur under the influence of extreme inanition, or after severe injuries with loss of blood, or towards the close of chronic exhaustive disorders, we do not find in them any marked liability to the infection of morbid poisons generally. In hospital practice, for instance, we do not find that typhus or erysipelas propagates itself among the patients of a ward in proportion to their weakness. It cannot be too distinctly understood that the predispositions to these various disorders are themselves as various as the disorders, and consist in specific conditions of blood, hitherto very imperfectly explored. Mere debility, as such, has nothing directly to do with the matter: a person is liable to the infection of small-pox, because he has one matter in his blood; to that of measles, because he has another; to that of typhus, because he has a third; to that of scarlatina, because he has a fourth, and so on. A predisposition to one of these disorders is by no means necessarily, and possibly not at all, a predisposition to any other of the class. As regards the exterior circumstances which are considered predisponent to infection, I apprehend there can be little question that their mode of operation must for the most part be indirect: that overcrowding and defective ventilation will increase the liability to small-pox or scarlatina only so far as they hinder a natural elimination from the blood of those materials which constitute the liability to the disease, or as they maintain those materials in a state of imperfect oxidation favorable to the zymotic change. In examining the habitations of the poorest classes in our large cities, we find the atmosphere highly animalised—often fetid with organic matters: the air is so little changed, that it stinks with the volatile excretions of the many human beings crowded together; and to these contaminations are very generally added products of decomposition arising from their other secretions, which lie in cesspools, or have soaked into the soil beneath and around their dwellings. Such an atmosphere can do little towards purifying the blood of matters wherewith itself is already so loaded: the effete matters of the organism, which naturally seek their elimination in an oxidised state, and which for the healthiest elimination ought probably to be in a high degree of oxidation, are here debarred from completing their discharge in its most normal form: and the inhabitants of such localities are consequently maintained artificially, replete with those humoral products which constitute the predisposition to zymotic blood-diseases. It is in these localities, if at all, that such diseases originate *de novo.* I entertain no doubt that some of them do thus originate: though I am unable to state what it is which gives the requisite impetus, or why it is given with one disease oftener than with another. Typhus appears so frequently to arise in this manner, and the predisposition to it so intimately associated with local circumstances, that some writers have been disposed to overlook the unquestionable evidence that exists of the re-production and multiplication of the poison in the person of the sufferer, and have inclined to consider it an enchorial disease, incommunicable by personal intercourse.

With respect to other alleged predisponent causes, I incline still more strongly to the view already expressed in regard of atmospheric influences, that they can operate only indirectly, only by means of those specific blood-products which constitute the true predisposition in each case. If fatigue predisposes to a particular infection, it can hardly be for any other reason than that the fatigued organ furnishes the material permissive of infection. If errors or insufficiencies of diet, or certain courses of medicine should be found to form a predisposition to certain infectible disorders, their mode of operation could scarcely be otherwise, than by increasing the formation, or diminishing the discharge of that blood product, which is the immediate object of attack to the zymotic poison.

Therefore, as respects those instances of human morbid infection with which we are best acquainted, we may recapitulate our facts, and state our theory, in the following terms: that certain materials of the blood—materials not essential to the performance of its nutritive functions, are, by certain circumstances, rendered liable to undergo definite and specific changes; under the influence of which they become determined, with increased rapidity, to the outlets of the body; and irritate these outlets in their passage; that these changes continue, until the materials affected by them are completely exhausted from the blood; and that the severity and duration of those changes is in proportion to the quantity of material seeking elimination: that the new matters engendered and evolved under these circumstances are capable in various ways, and with more or less certainty, of producing a precisely similar succession of changes in the blood of another individual, or of any number of individuals: operating always on some of the ingredients of the blood as that whence themselves arose, and determining it to the same outlets as that whither themselves were determined; so that the choice of material in the blood, and the choice of outlet in the body, constitute specific characters for the several morbid poisons distinctively, and so that the final products act always as special catalytics for that original material of the blood, wheresoever they may encounter it. But, finally, that under certain possible conditions of accumulation, or tension, in that original material, other circumstances may serve to start it in its progress of specific decomposition, without any demonstrable influence from that exterior catalytic, which is the ordinary occasion of its change.

From our foregone analysis of the pathology of morbid poisons, it is not difficult to deduce philosophical principles of treatment, or to devise a rational explanation of such success and such failure as medicine has hitherto encountered in this department of its ministrations. To check the further conversion of material into blood; to destroy the poison, or to turn it into harmless combinations; to aid or to anticipate the eliminative efforts of the disease; these would be the indications which pathology would suggest, and these have already, in great part, attained the sanction of experience. But both pathology and practice would concur in adding to these principles another, which, in our present age of palliative medicine, admits of almost infinite application; to remember (namely) that in each zymotic disease, nature is proceeding in her own way towards a curative termination, and that where (as too commonly happens) we are incompetent to conquer the disease by direct neutralizing antidotes, it behoves us chiefly to devote ourselves to the humbler task of moderating local phenomena, and sustaining constitutional power. Thus it is, that in a vast number of perilous infections, we are able to assist nature through her difficult process of cure, by no other treatment than the judicious administration of natural dietetic tonics—food and wine. Thus it is, that while we recognise the absolute efficacy of mercury against the poison of primary syphilis, we constantly find ourselves without an antidote against its later combinations, and confidently rely on measures adopted, without reference to the specific nature of the disease, solely on the ground of their general invigorating power. Till you can neutralise the poison of typhus, of erysipelas, of scarlatina within the blood of your patients, as you would neutralise an acid or an alkali in a test-tube, never lose sight of this important principle; never forget that these morbid poisons are eminently depressive to life; that they tend to kill by shock and debilitation.

And finally, see what vaccination has done for one of them, perhaps, formerly the most malignant and unsparing. I have taken pains to explain to you the pathology of its preventive power, and very little reflection on the argument of this lecture will convince you that there is, in the nature of things, no reason why small-pox alone should be frustrated in its tendency—no reason why each zymotic disease should not have its own preventive catalytic—no reason why, in connexion with these other pestilences, other men's names should not hereafter be remembered as gratefully and as gloriously as Jenner's in relation to small-pox. Our resources for this great purpose of preventive medicine are not restricted to the teats of cattle. We have the pharmacopœia before us, many of its articles acting catalytically on the blood, and determining products of decomposition, in a characteristic way, to a specific plurality of organs. Not only is there no reason against the possibility that many of these medicinal catalyses may be preventive of the zymotic catalyses; but there is every reason for such a possibility. To give you an illustration, why should not belladonna (determining the products of its operation to the throat, the kidneys, and the skin) act as a medicinal catalytic of that material which constitutes the susceptibility to scarlatina, and thus, in recognised reality (as heretofore in vague tradition) be preventive of that disease? Again, why should not the direct counteractive influence of drugs be extended in respect of these diseases, when they already are in attack? Why should we not be enabled by one drug to arrest the blood-change of typhus, and by another that of plague or glanders, just as with quinine we render the blood insusceptible of further detriment from the malarious poison.—*Lancet*, from *Braithwaite's Retrospect.*

